# Uncertainty About Geriatricians’ Roles Limits Access to Essential Care for Rural Veterans

**DOI:** 10.1093/geroni/igaf122.1229

**Published:** 2025-12-31

**Authors:** Eileen Dryden, Jessica Riley, Lauren Moo, Meaghan Kennedy, Camilla Pimentel, William Hung

**Affiliations:** Veterans Health Administration, Bedford, Massachusetts, United States; Veterans Health Administration, Quincy, Massachusetts, United States; VA Bedford Healthcare System, Bedford, Massachusetts, United States; VA Bedford Health Care System, Bedford, Massachusetts, United States; VA Bedford Health Care System, Bedford, Massachusetts, United States; James J Peters VA Medical Center, New York, New York, United States

## Abstract

Tele-health hub-and-spoke models, where urban specialists provide care for rural patients via telemedicine platforms, are one way to address the dearth of specialty care clinicians in rural areas. Two examples at the VA are GRECC Connect (GC), a program that provides rural patients access to multidisciplinary geriatrics teams, and Clinical Resource Hubs (CRH), a program that provides remote access to individual primary, specialty, and mental health clinicians. Both rely on clinicians making referrals to them for their older, medically complex patients. Built into this model is the assumption that referring clinicians understand what geriatricians do and place value on interdisciplinary geriatric care. As part of an ongoing evaluation of GC, between 2020-2024 we conducted qualitative interviews with directors, clinicians, and staff from 17 GC hub sites, four CRHs, and 13 referring clinics about these geriatric telemedicine services. We explored barriers and facilitators to establishing virtual geriatric services, promoting their uptake, and sustaining their use. While those that had used geriatric services for their patients generally found them beneficial, many potential referring clinicians and staff from ‘spoke’ sites seemed unsure what value geriatricians or geriatric teams would provide beyond their own knowledge. Given this, clinicians and staff from the CRH and GC ‘hubs’ provided substantial amounts of ongoing geriatric education and marketing to ‘spoke’ clinicians and staff to maintain use of their geriatric services. Promoting understanding of what geriatric services are and how they can benefit patients is essential for ensuring older, rural Veterans have access to evidenced- based geriatric services.

